# On the neuro-cognitive foundations of basic auditory number processing: an fMRI study

**DOI:** 10.1186/1744-9081-6-42

**Published:** 2010-07-09

**Authors:** Elise Klein, Korbinian Moeller, Hans-Christoph Nuerk, Klaus Willmes

**Affiliations:** 1 Department of Neurology, Section Neuropsychology, University Hospital, RWTH Aachen University, Germany; 2 Interdisciplinary Center for Clinical Research "BioMAT.", RWTH Aachen, Germany; 3 Institute of Psychology, Eberhard Karls University, Tuebingen, Germany

## Abstract

**Background:**

It is widely agreed that numbers automatically activate a magnitude representation. Nevertheless, so far no systematic evaluation of the neuro-cognitive correlates has been provided for the case of auditorily presented numbers.

**Methods:**

To address this question, we presented spoken number words in three different tasks (passive listening, magnitude comparison, parity judgement) as well as spoken pseudowords in an fMRI study.

**Results:**

We found IPS activation typically associated with magnitude processing in all tasks with numerical stimuli only. Interestingly, directly contrasting the two semantic tasks magnitude comparison (magnitude-relevant) and parity judgement (magnitude-irrelevant) revealed a left lateralized predominance within the IPS for the processing of parity information as compared to a right lateralization for number magnitude for auditorily presented number words.

**Conclusions:**

In summary, our results suggest a highly automatic activation of number magnitude for spoken number words similar to previous observations for visually presented numbers, but also indicate that the issue of hemispheric asymmetries deserves specific consideration.

## Background

Number magnitude and parity probably represent the two most important characteristics of natural numbers when classifications regarding the similarity of numbers have to be made (e.g., [1]). In this vein, magnitude comparison as well as parity judgement are among the most widely used tasks when investigating the mental representation of numbers. Nevertheless, although these two attributes are easily distinguishable for any number the representations of numerical magnitude and parity information do not seem to be mutually independent. It has been repeatedly shown that number magnitude representation is activated automatically even when magnitude information is not necessary to solve the task at hand [2,3] as e.g., in parity judgement (for a meta analysis and review see [4,5]; see also [6]; see [2] for fMRI data; but also see [7]). As a starting point for the current study, recent findings on both the neural correlate of number magnitude as well as parity information will be reviewed briefly.

### Investigating number magnitude representation

Generally, quantity information is supposed to be represented as analogue magnitudes aligned in ascending order along a nonverbal, logarithmically compressed, left-to-right oriented mental number line (e.g., [4,8,9]. On a neuro-functional level the (horizontal segment of the) intraparietal sulcus (hIPS) has repeatedly been identified to be vitally involved when it comes to processing number magnitude information of different formats (symbolic digits: e.g., [10,11]; dot patterns: e.g., [12,13], see [14] for common activations of the IPS for symbolic and non-symbolic quantity). In this context, it has been claimed that the representation of number magnitude is notation invariant, meaning that independent of the input format the same representation of numerical quantity is activated whenever one encounters any kind of numerical information (e.g., symbolic digits, dot patterns, etc. see [15,16]). Yet, to date mainly visual presentation of numerical stimuli has been employed to investigate number magnitude effects (but see [2] for fMRI data in a number identification task).

### Investigating parity representation

On the other hand, knowledge about the way in which number parity information is represented is much scarcer. Dehaene and co-workers [4] provided evidence for parity information to be retrieved from memory, thereby refuting other proposed strategies such as mentally dividing each to-be-classified number by 2 (cf. [17]). Moreover, when integrating behavioural and neuro-functional aspects into the Triple Code Model, Dehaene and Cohen [18,19] assumed that parity decisions may be closely linked to the identification of number symbols, a process suggested to be subserved by the so-called number form area (cf.[20]). Based on the pattern of impairment observed for patient NAU and the localization of his lesion, Dehaene and Cohen [21] assumed that it was the left number form area which was particularly involved in the processing of the parity status of a given number (see also [22]). Also, Plodowski and co-workers [23] observed an association of parity judgment with the number form area to be most pronounced in the Arabic digit condition when investigating parity judgements on written number words, dot patterns as well as Arabic and Roman number symbols in an EEG study. More recently, Iversen and colleagues [24] suggested that parity information may be accessed via two different routes. The authors made a distinction between language-specific and number-specific access to parity information depending on input format (i.e., linguistic vs. digital). However, this assumption by Iversen and colleagues [24] remained rather speculative so that neuro-functional evidence would be helpful to clarify the way in which number parity information is represented and accessed.

Interestingly, Plodowski et al. [23] as well as Iversen et al. [24] only used visually presented stimuli. Thus, as has been the case for number magnitude representation, the investigation of the representation of parity information has been largely limited to tasks involving visual presentation of stimuli so far (e.g. [21,4,23]).

Taken together, this short review illustrates the lack of studies directly investigating the interrelation of the two numerical representations of magnitude and parity information by employing neuro-imaging techniques. Currently, this issue is mainly addressed by behavioural studies (e.g. [4]) or lesion studies of brain damaged patients (e.g.[21]). However, almost all previous evidence on the interrelation of parity and magnitude representations relied on visually presented stimuli (e.g., [25]). As a consequence there is still only limited evidence for the case of auditory stimulus presentation. On a behavioural level, the observation of a reliable SNARC effect for auditorily presented stimuli suggests that the magnitude of auditorily presented numbers was activated automatically [26]. However, to the best of our knowledge, there are currently just very few studies evaluating the neural correlates of processing auditorily presented numerical stimuli (e.g.[2,27]). As the auditory presentation of numerical stimuli presents the specific aim of the current study, the latter studies will be discussed in the following.

### Neural correlates of processing auditory number words

First evidence regarding the neural correlates of auditorily perceived numbers comes from a study by Eger and colleagues [2]. In this study, the authors investigated the cortex areas activated by an identification task employing numbers, letters, and colours in both visual and auditory presentation mode. Functional imaging data revealed specific activation within the IPS which is generally assumed to subserve number magnitude processing (e.g. [28] for a review). Interestingly, such magnitude-related activation was observed for numerical stimuli even though number magnitude was irrelevant to solve the task (see also [29] for similar results for magnitude irrelevant tasks). Moreover, intraparietal activation was not modulated by input modality. From this it can be concluded that number magnitude representation may be accessed by both auditory and visual presentation of numerical stimuli. However, Eger and colleagues [2] came to this conclusion without explicitly contrasting IPS activation in a task for which magnitude information is relevant to IPS activation in a task for which magnitude information is irrelevant. Only data for the magnitude-irrelevant condition was obtained. One way of differentiating between magnitude-irrelevant and magnitude-relevant activation would be to contrast activation for parity judgement and magnitude comparison - as done in the current study. Furthermore, by employing passive listening tasks involving both spoken number words and spoken pseudowords we aimed at identifying more precisely, to which extent IPS activation reflects automatic processing of number semantics or may rather be associated with processing information not specifically related to numbers.

A first step towards a more comprehensive evaluation of magnitude relevant and irrelevant activation came from Wang et al. [27]. In their study, participants were assessed on both, calculation (magnitude relevant) and parity judgement (magnitude irrelevant) of auditorily presented numbers. For both tasks IPS activation was observed. However, as the study by Wang et al. [27] aimed at investigating the processing differences between first and second language, a direct comparison between calculation and parity judgement was not conducted.

Taken together, there is currently no systematic analysis evaluating possible differences in the neural correlates of processing auditorily presented numbers in either magnitude relevant or magnitude irrelevant conditions such as magnitude comparison and parity judgement, respectively. However, before elaborating on the specific objectives of the current study, a general issue about interpreting IPS activation shall be addressed.

### IPS activation due to processes of response selection and/or execution

The study by Cappelletti et al. [29] mentioned above is even more important for the present study in another respect: The authors aimed at dissociating IPS activation due to response selection processing from magnitude-related activation by partialling out effects of response time before evaluating activation differences between numerical and non-numerical conditions. Controlling for such response related effects seems to be important as Goebel and colleagues [30] have shown that response selection and number processing activate the same areas in the IPS. Thus, IPS activation in number magnitude comparison could be due to a mechanism for quantitative processing of numerical stimuli or it might be related to a general task component such as response selection or task difficulty [30-32]. Therefore, it cannot be excluded that common IPS activation for different input modalities in number identification tasks as employed by Eger et al. [2] might be partly due to response selection demands as argued by Cappelletti and coworkers [29]. Most studies investigating the effect of number magnitude on brain activations have not been able to exclude these alternative explanations. In this vein, Goebel et al. [30] even suggested that a strict dichotomy contrasting explanations based on response selection and number magnitude may not be appropriate since neural mechanisms of magnitude representation may be inextricably tied to response-selection mechanisms. According to Walsh [33] spatial and temporal magnitude representations in the parietal cortex are closely linked to response selection and the same has been proposed for the case of numerical magnitude. Even more importantly, Butterworth [34] referred to neuropsychological, developmental, and linguistic evidence to argue that numerical representation in the parietal cortex may be related to hand and finger based response processes in the same cortical area (see e.g., [35-39] for activation of cortex sites commonly associated with finger movement and number magnitude processing).

Taking into account these considerations the choice of passive mental tasks requiring no explicit, e.g., manual response, seems appropriate to keep activation related to response preparation and/or execution at a minimum. This approach, which is commonly used in neuroimaging (see e.g., [40-42] for the case of emotion processing) has already been utilized in the field of number processing (e.g., [32]). Since parity has also been found to be associated with response code properties (i.e., the linguistic Markedness Association of Response codes: even-right, odd-left), the choice of mental decisions seemed even more appropriate [43]. In summary, the paradigm without overt responses was chosen to obtain first neuro-imaging evidence on processing auditorily presented numbers at the same time minimizing confounding effects related to processes of response selection/execution, hand/finger movement, and/or associations with response codes.

### Objectives

In the present study, all stimuli were presented auditorily to systematically evaluate the activation pattern associated with auditorily presented numerical information. More specifically, we employed both non-numerical and numerical stimuli (magnitude relevant and magnitude irrelevant) to differentiate neural correlates associated with more or less automatic activation of the number magnitude representation.

We pursued this question using four mental numerical and non-numerical tasks and pink noise as a baseline condition:

(i) To establish a baseline condition, pink noise was presented with no explicit and/or implicit processing of numerical information. (ii) By using a passive listening task utilizing German number words we intended to avoid activation related to response selection. (iii) To evaluate whether or not the activation observed when listening to number words is indeed number-related, passive listening to German pseudowords was additionally used as non-numerical control task. (iv) A number magnitude comparison task was employed as an indicator for explicit number magnitude processing (for a review see [28]). (v) Finally, a parity judgement task was used to investigate the neural correlates of parity information processing in auditorily presented one-digit numbers. The range of tasks/questions allowed for minimizing differences in the type of information that was extracted from number words and pseudowords.

Two main questions were examined: (i) What is the typical activation pattern for the processing of auditorily presented number words? (ii) Is it possible to identify differences in localization and/or intensity of fMRI signal change due to the processing of either magnitude relevant tasks (such as number magnitude comparison) or magnitude irrelevant tasks (such as parity judgement)? Taking into account the results of Cappelletti et al. [29] allowed for a specification of this hypothesis: these authors observed common bilateral IPS activation for conceptual decisions on numbers, but more specific analyses revealed a left-hemispheric lateralization for extraction of learned (possibly verbally stored) information on numbers (e.g., deciding whether a date is in summer), while the right IPS was reliably more involved in more specific processing of numerical information (e.g., magnitude comparison). Extrapolating these results to the present study led us to assume common bilateral IPS activation for both magnitude comparison and parity judgement. However, the direct comparison of the two tasks should reveal more right hemispheric IPS activation for the number magnitude comparison task. On the contrary for parity judgement we suggested more pronounced left-hemispheric IPS activation, because the left IPS may be involved in the extraction and comparison of learned information.

The corresponding analyses were conducted in three consecutive steps starting from checking whether participants indeed processed the auditorily presented numbers in the respective tasks to much more specific contrasts investigating possible differences and similarities of the neural correlates of a magnitude relevant number comparison task and a magnitude irrelevant parity judgement task:

In the first step, all tasks were contrasted to the baseline condition (i.e., pink noise). Generally, for the current auditory presentation of German number words we expected to observe IPS activation typically associated with number processing. However, the respective comparisons also allow for more specific inferences to be drawn: (a) The contrast of passive listening to number words vs. pink noise should indicate activation related to (implicit) magnitude processing not contaminated by processes of response selection. (b) Contrasting number magnitude comparison to pink noise should specifically yield magnitude related activation to auditory stimuli. Moreover, we reasoned that when intraparietal activation due to passive listening to number words is already driven by quantity processing, then IPS activation should be higher for the magnitude comparison task as it requires explicit processing of number magnitude information. (c) Comparing parity judgement to pink noise should be informative regarding the neural correlates of parity information processing when being presented auditorily.

In the second step, passive listening to number words was contrasted to passive listening to pseudowords to isolate activation reflecting task irrelevant automatic magnitude processing. This analysis was used to clarify whether the contrasts between the numerical experimental conditions and the pink noise baseline reveal specific information about the processing of numerical magnitude instead of other potential variables, such as differences in attention deployed to meaningful (number words) versus meaningless (pseudowords) auditory stimuli.

Finally, in the third and most specific step the task specific activation for magnitude comparison and parity judgement were directly contrasted. Thereby, we aimed at investigating differences in the activation patterns for magnitude comparison and parity judgement.

Taken together, the research questions driving the current study were threefold: (i) What is the activation pattern associated with the processing of auditory numerical stimuli? (ii) Can activation related to (implicit) magnitude processing be found whenever the auditory stimulus contains numerical words, irrespective of whether there is no task at hand or the task at hand is asemantic numerically? (iii) Are there any differences and/or similarities in localization and/or intensity of fMRI signal change specifically associated with either magnitude comparison (i.e., more right IPS activation) and/or parity judgement (i.e., more left IPS activation)?

## Methods

### Participants

17 male German-speaking students participated in the current study (mean age: 24.9 years; SD = 1.7 years). All participants were right-handed and reported no neurological or arithmetic impairment. Participants were recruited on a volunteer basis and provided their written informed consent in accordance with the protocol of the Ethics Committee of the Medical Faculty of the RWTH Aachen University. One participant had to be excluded from the analysis due to technical problems in recording the imaging data.

### Stimuli and Design

As the current study was part of a larger study, stimulus material comprised German pseudowords, pseudowords similar to number words, and German number words ranging from 0 to 9, all of them obeying standard German phonotactic constraints. Corresponding pairs of stimuli (i.e., pseudowords and number words) were created such that their number of syllables and phonemes as well as, for the only bisyllabic pair ("sieben" (seven) vs. "leumer"), also the segmental structure was identical (see Table 1). All stimuli were spoken by an experienced male speech therapist and recorded with the support of the Audio-Visual Media Centre at the University Hospital Aachen. To minimize possible influences of low-level auditory features such as pitch, loudness, or accent these were held constant. This approach was chosen to decrease phonetic variation between German number words and pseudowords. All stimuli were presented auditorily via headphones.

**Table 1:** List of auditory stimuli used. Stimulus material consisted of German number words and German pseudowords obeying German phonotactic constraints.

Arabic Numbers	Number words	Pseudowords	Number of phonemes	Number of syllables
0	null	nitz	3	1
1	eins	dosch	3	1
2	zwei	muug	3	1
3	drei	seub	3	1
4	vier	jees	3	1
5	fünf	knahr	4	1
6	sechs	gooft	4	1
7	sieben	leumer	5	2
8	acht	niff	3	1
9	neun	bauf	3	1

Number of phonemes and syllables were matched for number words and related pseudowords.

The experiment was conducted in a box-car design with 8 runs, each requiring participants to perform another task with the presented words. (i) Passive listening: In the first three runs participants had to listen passively to spoken German pseudowords, pseudowords similar to number words and finally spoken number words, respectively. (ii) Phoneme detection: In the next three runs participants had to decide mentally whether or not the spoken stimuli presented (again in the order pseudowords, pseudowords similar to number words, and number words) contained the phoneme /f/ or /s/. This was the case for half of the stimuli. (iii) Parity judgement: In the seventh run, participants were asked to decide mentally whether the presented number word corresponds to an even (i.e., 0, 2, 4, 6, 8) or an odd number (i.e., 1, 3, 5, 7, 9). (iv) Number Comparison: In the last run only the numbers from 1 - 4 and 6 - 9 were presented and participants were supposed to mentally compare the magnitude of the number word to the fixed standard of 5.

As the current study aimed at investigating the interrelation of the representations of number magnitude as well as parity information, data acquired in the phoneme detection tasks as well as in runs using words similar to number words were not considered in the subsequent analyses. Exactly the same auditory stimuli were used for all tasks on number words meaning that for passive listening, parity judgement as well as magnitude comparison the same number word recordings were presented. This ensured that possible activation differences between that set of tasks cannot be attributed to differences in low-level auditory features. Instead, this approach should allow for identifying different levels of semantic processing.

Run order was not randomized so that all participants had to perform the two semantic numerical tasks (i.e., parity judgement and magnitude comparison) last. Thereby, activation in the non-semantic tasks (i.e., passive listening) was not influenced by preceding tasks requiring semantic evaluation of numerical information.

Each run consisted of an off-on-off-on-sequence lasting for 180 seconds. Every on-phase comprised 20 spoken number words, whereas in every off-phase a neutral sound (pink noise) was presented which was identical to the spoken numbers with regard to frequency, modulation, and duration. Activation during these off-phases served as a baseline condition. Trial order was pseudo-randomized to preclude any systematic confounding between condition and stimulus order as each participant was presented the same sequence of trials. Additionally, trial order was chosen with the constraint that the same decision (e.g., a magnitude larger than five in the number comparison task) was not to be made more than 3 times in a row. Each run started with a different sequence of words to prevent any expectation regarding the number following. Each word was presented twice per on-phase.

### Procedure

Participants were lying in the scanner and listening to the stimuli presented auditorily via headphones. Participants had to perform the active tasks (i.e., number comparison and parity judgement) mentally without giving any overt response. Since IPS activation has also been shown to be associated with processes of response preparation [30] as well as hand and finger based response processes [34], a mental decision paradigm was chosen to keep activation related to response preparation and/or execution at a minimum (for further details please see the Discussion). Head movements were restricted to a minimum by soft foam pads positioned between the head of the participants and the head coil. Participants were given the instructions for the first run before entering the scanner. Any further instructions were presented via headphones before starting the respective run.

### Scanning procedure and data acquisition

One functional imaging run sensitive to blood oxygenation level-dependent (BOLD) contrast was recorded for each participant with the Philips 1.5T Gyroscan MRI system (T2*-weighted echo-planar sequence, TR = 3000 ms; TE = 30 ms; flip angle = 90°; FOV = 225 mm, 64 × 64 matrix; 35 slices, voxel size = 3.5 × 3.5 × 3.5 mm, no gap). The experimental trials were presented in a box-car design at a mean rate of one trial every 2.25 seconds. Auditory presentation of the number words lasted from 800 - 1000 ms. In one run, 48 scans were acquired (60 × 3 seconds (TR) covered about 180 seconds). Two initial dummy scans, which were not recorded for data analysis, were used to establish equilibrium magnetization.

The fMRI time series was corrected for movement artefacts and unwarped in SPM2. Images were motion corrected and realigned to each participant's first image. Data were normalized into standard stereotaxic space. Images were resampled every 3.5 mm using standardized interpolation and smoothed with a 7 mm FWHM Gaussian kernel to accommodate inter-subject variation in brain anatomy and to increase signal-to-noise ratio in the images. The data were high-pass filtered (128 s) to remove low-frequency signal drifts and corrected for autocorrelation assuming an AR(1) process for the time-series of data. Brain activity was convolved over all experimental trials with the canonical hemodynamic response function (HRF). Localization of activation peaks was determined using the Co-Planar Stereotaxic Atlas of the Human Brain by Talairach and Tournoux [44] as well as the Talairach Daemon Client [45].

Complex contrasts were generally masked inclusively making sure that in a complex contrast (e.g., A - B) only activation in those voxels gets displayed which were already activated (not deactivated) in the minuend (here: A). In order to display the results of analyses consistently, we generally chose a cluster size of k = 10 voxels.

## Results

Analysis of fMRI data was based on all trials. As mentioned above, the analyses were conducted in three consecutive steps. The description of the results will follow these steps. To pursue our first goal of evaluating whether the tasks employed in the present study elicited magnitude-related activation when they were presented auditorily, the activation pattern observed in each individual task was contrasted to the activation of the control task.

**Figure 1:**
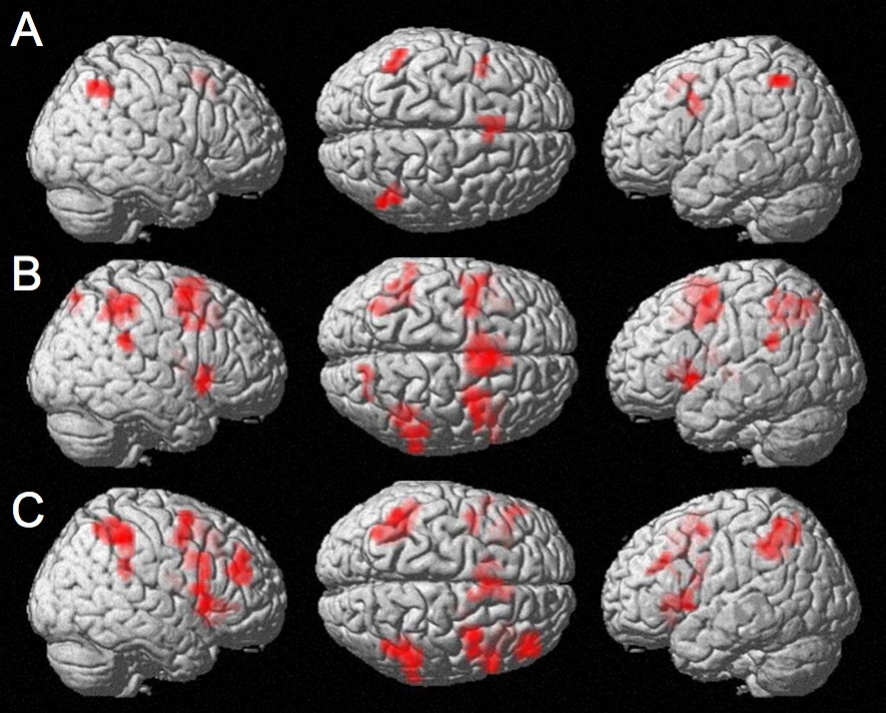
**Intraparietal activation during tasks with numerical stimuli. **A: Passive listening - control at an uncorrected voxelwise p < .0001 and cluster size k = 10 voxels, masked inclusively with passive listening: Magnitude-related activation for number words which cannot be due to response selection. B: Number comparison - control at an uncorrected voxelwise p < .0001 and cluster size k = 10 voxels, masked inclusively with number comparison: Magnitude-related activation for auditory stimuli - similar to the activity previously reported for visual stimuli. C: Parity - control at an uncorrected voxelwise p < .0001 and cluster size k = 10 voxels, masked inclusively with parity: Activation of the bilateral IPS is observed.

Passive listening to number words vs. baseline condition: Passive listening to numbers was contrasted to pink noise at an uncorrected voxelwise *p *< .0001. This comparison already indicated magnitude-related activation in the bilateral intraparietal sulci [Brodmann Area (BA) 7 and BA 40], although no response selection was required in either task (see Figure 1A, Table 2). Further clusters of activated voxels were found in the left middle frontal (BA 9) and superior frontal gyri (BA 6).

**Table 2:** Magnitude-related activation for all four tasks when presented auditorily.

Contrast	Brain region (BA)	TC (x, y, z)			Cluster size	Z score
Passive listening -	LH intraparietal sulcus (BA 7)	-45	-52	51	18	4.87
Pink noise baseline	RH intraparietal sulcus (BA 40)	49	-49	44	30	4.55
	LH middle frontal gyrus (BA 9)	-49	12	35	17	4.22
	LH superior frontal gyrus (BA 6)	-7	9	48	35	4.48
						
Magnitude comparison -	RH intraparietal sulcus (BA 7)	38	-42	44	96	5.03
Pink noise baseline	LH intraparietal sulcus (BA 40)	-49	-42	41	71	4.73
	RH precuneus (BA 7)	10	-72	49	21	4.50
	RH supramarginal gyrus (BA 40)	66	-36	24	17	5.53
	LH supramarginal gyrus (BA 40)	-52	-43	25	24	5.19
	RH medial frontal gyrus (BA 6)	3	3	55	230	5.63
	LH middle frontal gyrus (BA 6)	-31	-1	42	97	5.27
	RH middle frontal gyrus (BA 6)	28	-1	45	92	5.25
	RH middle frontal gyrus (BA 8)	45	12	35	22	4.79
	LH insula	-42	10	-1	54	5.28
	RH insula	38	17	2	69	4.83
	LH thalamus	-10	-10	1	32	4.62
						
Parity -	RH intraparietal sulcus (BA 7)	42	-42	44	114	6.75
Pink noise baseline	LH intraparietal sulcus (BA 40)	-38	-42	41	156	5.53
	RH supramarginal gyrus (BA 40)	62	-36	28	15	4.51
	RH middle frontal gyrus (BA 46)	45	35	18	72	4.98
	LH middle frontal gyrus (BA 46)	-45	29	28	23	4.68
	RH inferior frontal gyrus (BA 44)	49	11	9	173	5.31
	RH inferior frontal gyrus (BA 47)	38	21	2		
	LH superior temporal gyrus (BA 22)	-49	10	-1	19	4.87
	LH cingulate gyrus (BA 32)	-14	15	35	16	5.73
	LH superior frontal gyrus (BA 6)	-3	9	48	94	5.63
	RH middle frontal gyrus (BA 6)	45	6	55	64	5.65
	LH middle frontal gyrus (BA 6)	-38	5	29	14	5.51
	LH precentral gyrus (BA 6)	-45	2	45	13	4.81
	LH insula	-31	14	3	48	5.61
	RH lentiform nucleus	21	-3	16	10	4.48

p < .0001, uncorrected; cluster size = 10 voxels; masks were created at uncorrected p < .05; TC - Talairach coordinates.

Number comparison vs. baseline condition: Contrasting number comparison and pink noise baseline (*p *< .0001, uncorrected) revealed magnitude-related activation in the bilateral intraparietal sulci (BA 7 and BA 40), which corresponds to magnitude-related activity previously seen in the number comparison task with visual stimulus presentation. Furthermore, task specific activation was also observed in the right precuneus (BA 7), the bilateral supramarginal gyri (BA 40), the bilateral middle frontal gyri (BA 6) and the right medial frontal gyrus (BA 6) (Figure 1B, Table 2). Further clusters of activated voxels were found in the bilateral insula and the left thalamus.

Parity judgement vs. baseline condition: Parity was contrasted with pink noise baseline at an uncorrected voxelwise *p *< .0001. Again, activated voxels were observed in the bilateral intraparietal sulci (BA 7 and BA 40). Further maxima of activation were found in the right supramarginal gyrus (BA 40), the bilateral dorsolateral prefrontal cortices (DLPFC, BA 46), the right inferior frontal gyrus (BA 44, extending into BA 47), the left superior temporal gyrus [Wernicke's area (BA 22)], the left cingulate (BA 32), superior frontal (BA 6) and precentral gyrus (BA 6), the bilateral middle frontal gyri (BA 6), the left insula, and the right lentiform nucleus (Figure 1C, Table 2). Despite this wide-spread activation pattern no activation could be observed in the visual number form area.

In a second step, passive listening to number words was compared to the non-numerical control task passive listening to pseudowords to evaluate to what extent the intraparietal activation during the tasks with numerical stimuli indeed involved access to the representation of numerical magnitude rather than non-numerical factors such as attention and covert response selection.

**Figure 2:**
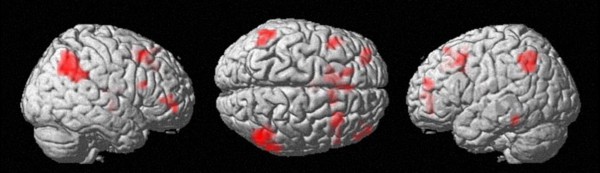
**Comparing a numerical with a non-numerical task. **Passive listening to number words - passive listening to pseudowords at an uncorrected voxelwise p < .005 and cluster size k = 10 voxels: Magnitude-related IPS activation in auditory processing

Passive listening: number words vs. pseudowords: Contrasting passive listening to number words with passive listening to pseudowords (*p *< .005, uncorrected) yielded activation in the bilateral intraparietal sulcus (BA 40), extending in the right hemisphere into the posterior intraparietal sulcus (BA 40) and in the left hemisphere into the supramarginal gyrus (BA 40, Figure 2, Table 3). Further clusters of activated voxels were observed in the left superior temporal gyrus (BA 22), in the bilateral cingulated gyri (BA 32), the bilateral middle frontal gyri (BA 46, BA 10, BA 8), the right thalamus, and the left medial frontal gyrus (BA 8).

**Table 3:** Comparing a numerical with a non-numerical task to isolate magnitude-related activation.

Contrast	Brain region (BA)	TC (x, y, z)			Cluster size	z score
Passive Listening:	RH intraparietal sulcus (BA 40)	49	-56	32	137	3.96
Number words - pseudowords	RH posterior intraparietal sulcus (BA 40)	49	-59	48		3.57
	LH intraparietal sulcus (BA 40)	-55	-45	47	51	3.77
	LH supramarginal gyrus (BA 40)	-59	-52	38		3.58
	LH middle frontal gyrus (BA 46)	-31	45	17	37	3.61
	LH superior temporal gyrus (BA 22)	-55	-41	-13	10	3.51
	LH cingulate gyrus (BA 32)	-17	15	31	13	3.51
	RH cingulate (BA 32)	14	19	41	14	3.20
	RH middle frontal gyrus (BA 10)	45	51	-5	18	4.03
	RH middle frontal gyrus (BA 8)	35	19	41	23	3.39
	LH middle frontal gyrus (BA 8)	-45	19	41	18	3.35
	RH thalamus	7	-6	13	17	3.30
	RH thalamus	10	-20	14	13	3.22
	LH medial frontal gyrus (BA 8)	-7	19	44	41	3.20

p < .005, uncorrected; cluster size = 10 voxels; masks were created at uncorrected p < .05; TC - Talairach coordinates.

Taken together, by contrasting a numerical task (passive listening to number words) to a non-numerical task (passive listening to pseudowords), which involves auditory stimuli that do not contain any numerical information, activation reflecting numerical processing could be isolated.

Finally, in the third and last step, the activation patterns indicating task specific processing in magnitude comparison and parity judgements were directly compared to investigate possible differences between number comparison and parity judgement.

**Figure 3:**
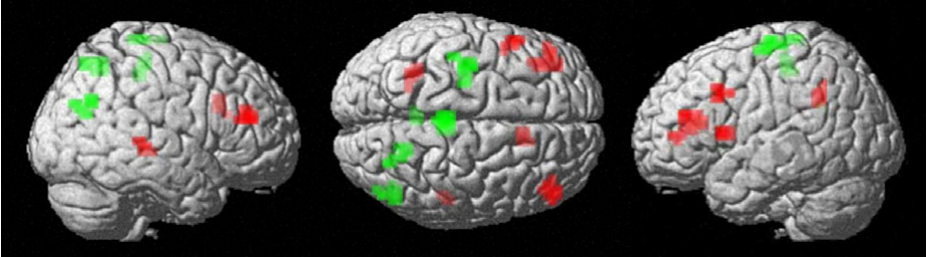
**Task specific processing in magnitude comparison and parity judgement. **Superimposed contrasts (uncorrected voxelwise p-value of < .005, cluster size k = 10 voxels): Red voxels indicate number comparison - parity, each contrasted to the pink noise baseline condition; green voxels indicate parity - number comparison. Processing of parity information relies relatively more on the left-hemispheric IPS.

Number comparison vs. parity judgement: Number comparison was contrasted to parity at an uncorrected voxelwise *p *< .005, both tasks with respect to the pink noise baseline (Figure 3, Table 4). Activated voxels were found in the right posterior intraparietal sulcus (BA 7), the right angular gyrus (BA 39), and the right paracentral lobule (BA 4).

**Table 4:** Comparing only response-selective tasks: Specificity for representations?

Contrast	Brain region (BA)	TC (x, y, z)			Cluster size	z score
Magnitude Comparison -	RH posterior intraparietal sulcus (BA 7)	17	-55	48	20	3.73
Parity	RH angular gyrus (BA 39)	52	-60	29	15	3.36
	LH precentral gyrus (BA 4)	-35	-14	62	18	3.12
	LH paracentral lobule (BA 4)	0	-27	66	16	3.40
						
Parity -	LH intraparietal sulcus (BA 40)	-31	-49	31	18	3.74
Magnitude Comparison	LH inferior frontal gyrus (BA 45)	-38	28	15	33	3.57
	LH inferior frontal gyrus (BA 44)	-52	11	9	13	3.50
	LH middle frontal gyrus (BA 9)	-52	15	35	10	3.34
	RH middle frontal gyrus (BA 46)	42	35	18	19	3.27
	RH anterior cingulate gyrus (BA 24)	10	21	21	12	3.42
	RH superior temporal gyrus (BA 22)	49	-30	5	14	3.53

p < .005, uncorrected; cluster size = 10 voxels; masks were created at uncorrected p < .05; TC - Talairach coordinates.

Parity judgement vs. number comparison: Comparing parity to number comparison at an uncorrected voxelwise *p *< .005, activation was found in the left intraparietal sulcus (BA 40). Further clusters of activated voxels (Figure 3, Table 4) were observed in the left inferior frontal gyrus (Broca's area, BA 44 and 45), the bilateral dorsolateral prefrontal cortex (BA 9 and BA 46), the right anterior cingulated gyrus (BA 24), and the right superior temporal gyrus (BA 22).

In summary, when presenting numbers auditorily, both, magnitude comparison as well as parity judgement seemed to rely on access to the quantity representation of numbers as reflected by bilateral intraparietal activations. Moreover, a direct comparison between magnitude comparison and parity judgement revealed fine-grained differences in lateralization and location: Whereas the right posterior intraparietal cortex is significantly more involved when performing the auditory magnitude comparison task, performance in the auditory parity judgment task revealed significantly more activation of the horizontal segment of the left intraparietal sulcus.

## Discussion

The current study set off to investigate three main questions regarding the processing of auditorily presented number words. First, it was observed that the activation pattern associated with the processing of auditorily presented number words mainly involved the bilateral intraparietal sulcus (IPS) - a cortex site generally assumed to and typically found to be activated by number processing (for a review see [28]). More specifically, as regards the interrelation of parity judgment and number magnitude comparison, particular interest was paid to possible differences and/or similarities in localization and/or intensity of fMRI signal change associated with either magnitude comparison and/or parity judgement, thereby, adding to our understanding of the representation of parity information. However, before discussing these two aspects in turn, the general validity of our results will be illustrated.

### Validity of the current results

The present study aimed at evaluating the neural correlates of number magnitude comparison and parity information. Therefore, it was intended to discern IPS activation related to magnitude processing from IPS activation induced by other processes such as response selection/execution [29,32,30], hand and finger movement [35,37], and/or associations with response codes [43]. To do so, we presented a systematic combination of passive tasks (numerical and non-numerical stimulus material) and numerical tasks with mental decision (magnitude relevant and magnitude irrelevant) to keep activation related to response preparation and/or execution at a minimum. However, as no manual responses were recorded the question arises whether participants indeed performed the tasks as requested - a necessary precondition for the validity of our data and the conclusions we are about to draw.

Evidence for the validity of our data comes from several different aspects. First, the different localizations and intensities of the fMRI signal observed for the different tasks indicated that participants indeed performed different mental tasks. More detailed investigation of the activation patterns revealed that mental processes involved are most likely task and stimulus dependent. By using a number magnitude comparison task we were able to replicate the activation pattern typically associated with number magnitude processing with manual responses for the case of mental decisions (for a review see [28]). This observation indicated that the present data are generally in line with previous neuro-cognitive data on the representation of number magnitude and their theoretical conceptualization in recent models of number processing. Moreover, IPS activation was stronger and spread out over a larger number of voxels for the magnitude comparison task as compared to e.g., listening passively to number words which is also supposed to automatically activate the quantity representation (e.g., [2]). This finding again supports the assumption that in the mental decision tasks (here magnitude comparison) participants indeed executed additional mental processes related to processing the semantics of the presented numbers. Finally, passive listening to pseudowords was associated with activation of a different neural network (i.e., not including the IPS) than listening to number words. Once again, this suggests that the contrasts between the numerical experimental conditions may indeed reveal specific information about the processing of number semantics such as magnitude and/or parity information. These results also mirror recent results from other studies on numerical cognition not using manual responses (e.g., [32]) for the case of visually presented stimuli.

Another important aspect regarding validity of the current results may be the fact that the different tasks were presented in a fixed order. The rationale behind this ordering was to minimize a possible bias towards semantic processing of the presented stimuli in non-semantic tasks (e.g., passive listening) by the fact that semantic magnitude information was critical in the preceding run. However, because passive listening was always presented prior to the parity task which was in turn followed by the magnitude comparison task we cannot and do not want to exclude that this might in turn have influenced the current results to some degree. In particular, one might speculate whether the observed lateralization for the parity task activation may have been influenced by presentation order. Although we cannot reject this possibility entirely, we are confident that left lateralization of the parity judgment task is not a mere artefact produced by the order of tasks but indeed adds to our knowledge about the specificities of processing different aspects of numerical information. In this context it is important to acknowledge that left lateralization of activation associated with language-based numerical information such as parity judgement has been reported only recently by Cappelletti and colleagues ([29]) and has also been suggested by a prominent model of numerical cognition ([18]). Nevertheless, we think that it is important to address the issue of possible order effects in numerical cognition more thoroughly in future studies to better understand the interactivity of human numerical cognition.

Taken together, the current paradigm seems to be a valid approach to further investigate the neural correlates of processing auditory number words with minimizing concurrent IPS activation due to processes of response selection/execution, hand/finger movement and/or associations with response codes. The implications of the current result will be evaluated in the following paragraphs.

### Generalizability of neural correlates

As repeatedly observed for number magnitude processing (see [28] for a review), reliable bilateral IPS activation was observed for all contrasts employing numerical stimulus material (i.e., number comparison, passive listening to number words, and parity judgement) as opposed to non-numerical stimulus material (pseudowords). These observations are in line with the assumption of a supramodal representation of number within the human intraparietal sulcus as proposed by Eger et al. [2].

Furthermore, there are several aspects suggesting that the observed intraparietal activation reflects number magnitude processing in all instances, even when passively listening to number words. First, on a theoretical level, it is assumed that in the context of number processing IPS activation reflects the processing of number magnitude information [28,9]. Second, inspection of the fMRI results indicated that even when magnitude was not necessary to solve the task - as is the case in parity judgements - magnitude-related IPS activation was found. This is in line with previous observations for visually presented numerical stimuli which suggested that number magnitude is activated even when it is not relevant for the task at hand ([4], see [2] for fMRI data). Moreover, the contrast of passive listening vs. the baseline condition (pink noise) indicated that even in the absence of any numerical task and consequently in the absence of any need for response selection, number magnitude seems to be automatically activated as reflected by significant IPS activation (see Step 1 analyses). This interpretation is backed-up by the results of the contrast between passive listening to number words versus pseudowords indicating magnitude-related IPS activation only for passive listening to number words. This means that merely listening to auditory stimuli does not seem to result in obligatory intraparietal activation. Instead, these findings corroborate the assumption of automated processing of number magnitude information even when presented auditorily.

To our knowledge this is the first time that such magnitude-related activation has been reported for passive listening to spoken number words. Moreover, based on the paradigm requiring mental decisions only, this magnitude-related activation should not be confounded by other processes such as response-selection or hand and/or finger movement. This is of particular importance for the interpretation of the IPS activation patterns in the numerical tasks (i.e., magnitude comparison, parity judgement): When a voxel was activated for the passive listening task (requiring no response) and for e.g., the parity judgement task (requiring a mental response) it is very unlikely that the involvement of this particular area in the parity judgement task can be attributed to processes of response selection.

### The interrelation of parity and number magnitude information

In line with previous studies using visual presentation, the auditorily presented parity judgement task was associated with bilateral IPS activation ([27]; see [46] for a TMS approach). However, as argued above, this observation did not allow for an identification of neural activation specific to the processing of parity information: Since bilateral IPS activation was found even for passively listening to spoken number words, activation devoted to the processing of parity information cannot be separated form activation elicited by the automatic processing of number magnitude. However, specifics of the judgement of parity information may still be identified by evaluating possible differences between the neural correlates of magnitude comparison and parity judgement. As Figure 3 depicts activation associated with parity judgement was stronger in the left hemisphere when compared to number comparison. Additionally, Figure 3 also shows that this pattern was reversed for magnitude comparison, for which activation was more pronounced in the right hemisphere. Thus, this hemispheric asymmetry provided evidence for a relative lateralization in the processing of number magnitude and parity information.

On the one hand, the right lateralized activation observed for magnitude comparison is in line with previous findings suggesting a right hemispheric predominance for the processing of number magnitude (e.g., [47-49], but see [10]). More particular, this lateralization fits very well with recent findings by Cappelletti et al. [29] who observed number-selective right hemispheric IPS activation during both quantity and non-quantity tasks involving numbers. Moreover, this number-selective activation was present even when controlling for task and response related effects. On the other hand, the authors suggested that left IPS activation in numerical tasks may reflect more general processes of the extraction and comparison of learnt information [29]. In this vein, the more pronounced activation for parity judgements found in the left hemisphere may indicate a "direct retrieval of parity information from a semantic store of simple arithmetical properties" ([4] p. 393; [50]). Within the theoretical framework of the Triple Code Model [18,19,28] this store of numerical properties is assumed to be located in left hemispheric perisylvian language areas. According to Cappelletti et al. [29] the left IPS may also be more engaged in the exact processing of symbolic, language-based numerical information. In particular, the authors suggested left intraparietal regions to subserve the extraction of information from numerical symbols in order to retrieve the exact representation of symbolic numbers. Therefore, the left lateralized activation for parity judgement may reflect additional retrieval of parity information not primarily necessary in magnitude comparison. The association of stored arithmetical properties with left hemispheric language areas also drives the assumption of this store being verbally mediated.

The idea that parity retrieval may be (also) linguistically mediated is not totally new. At this point the linguistic Markedness Association of Response Codes (MARC) effect comes into play [[43]; see also [51,24]). Within this context, processing of parity information (i.e. "even" or "odd") is influenced according to the concept of linguistic markedness, a basically verbal attribute of (number) words ([52,53]; see [43] for a more detailed discussion). Conceptualizing the parity of a number as a linguistic/verbal attribute of the corresponding number word, this is also in line with the observation of more pronounced activation for parity judgement in the left hemisphere. Furthermore, this finding corroborates the assumption of Iversen and colleagues [24] that there may be two different routes to number parity information: a language-specific route, on the one hand, and another route providing number-specific access to parity information, on the other hand. In the light of this proposition, the left lateralized increase in activation for parity judgement observed in the current study might indicate that the route to parity from linguistic input (as spoken number words are) may be left lateralized.

## Summary and Conclusions

The current study presents a first step towards a more comprehensive understanding of the processing of auditorily presented number words leaving further steps to be taken. In particular, it would be desirable for future studies to investigate this issue by directly comparing the processing of visual and auditory input. Nevertheless, the results of the current study are meaningful as they indicate that activation of number magnitude information for auditory number words seems to be quite similar to activation typically reported for the processing of numerical stimuli. IPS activation commonly associated with number magnitude was observed in all conditions using numerical stimulus material: In particular, it was obtained not only in the semantic magnitude-relevant magnitude comparison task, as well as in the also numerical, but magnitude-irrelevant parity judgement task, but also in the passive listening task to number words in which no response selection was required at all. Contrarily, no intraparietal activation was observed when participants had to passively listen to pseudowords suggesting a highly automatic activation of number magnitude in humans for spoken number words similar to previous observations for visually presented numbers (see [28] for a review).

More particularly, the present study provides a first systematic evaluation of the neural correlates and the interrelation of associated representations of numerical magnitude and parity information when stimuli are presented auditorily. First, bilateral activation of the IPS in both tasks indicated that the assumption of automatic activation of the magnitude of an encountered number generalized to auditory stimuli. Second, a left lateralized predominance within the IPS for the processing of magnitude irrelevant parity information for auditorily presented number words, as compared to a right lateralization for number magnitude, corroborates the notion that parity information may be represented in some kind of verbally mediated store for simple arithmetical properties. Finally, this left lateralization of the mental representation of parity information for auditory stimuli raises the question whether hemispheric asymmetries in numerical cognition may be more pronounced when stimuli are presented auditorily.

## Competing interests

The authors declare that they have no competing interests.

## Authors' contributions

KW and HCN conceived the study. All authors participated in its design. EK performed data collection, processing and statistical analyses. EK and KM drafted the manuscript; the other authors revised it critically. All authors contributed to the interpretation of the data. All authors read and approved the final manuscript.
